# The prevalence of hepatitis B virus E antigen among Ghanaian blood donors

**DOI:** 10.11604/pamj.2014.17.53.3390

**Published:** 2014-01-24

**Authors:** Tanko Rufai, Mohamed Mutocheluh, Kwaku Kwarteng, Elliot Dogbe

**Affiliations:** 1 Department of Clinical Microbiology, School of Medical Sciences, Kwame Nkrumah University of Science & Technology, Kumasi, Ghana; 2 Komfo Anokye Teaching Hospital, Kumasi, Ghana

**Keywords:** Prospective blood donors, HBeAg, HBsAg, Hepatitis B virus, Hepatocellular carcinoma, Ghana

## Abstract

Hepatitis B viral infection is an important clinical problem due to its worldwide distribution and potential of adverse sequelae, including hepatocellular carcinoma (HCC). We studied the prevalence of hepatitis B virus ‘e’ antigen (HBeAg) among individuals determined to be hepatitis B virus (HBV) surface antigen-positive and analyzed the gender/age category associated with more active HBV infection and whether alteration in the levels of alanine aminotransferase could be associated with HBeAg positivity. A total of 150 prospective blood donors who tested positive for hepatitis B surface antigen (HBsAg) at the blood transfusion center of the Komfo Anokye Teaching Hosptital (KATH), Kumasi were randomly selected for the study. The serum samples were further tested for HBsAg and HBeAg using a lateral flow immunochromatographic assay. Twenty (20) individuals were found to be HBeAg-positive giving an overall prevalence of 13.3%, of which 18 (15.5%) were males and 2 (5.9%) were females. Our results also revealed that the prevalence of HBeAg was higher in patients between the age group of 10-20 years and appeared to decrease with increase in age. There was no statistical difference between the HBeAg positive and negative individuals with respect to alanine aminotransferase (ALT) levels. We show for the first time that approximately 1/10 of HBV-infected individuals are HBeAg positive in the Ashanti Region of Ghana, suggestive of active viral replication and liver-cell infectivity thereby contributing to an increased HBV-transmission pool within the Ghanaian population.

## Introduction

The world health organization estimates that over 2 billion people have been exposed to hepatitis B virus and approximately 350 million people are chronically infected with HBV [1]. In Ghana HBV prevalence is estimated at >10% among blood donors, increasing in the rural populations [2].

Chronic HBV infection is the risk factor for the development of HCC [3]. The progression of chronicity and the associated pathogenesis directly correlate with active viral replication demonstrable by serological markers [4]. HBeAg is one of the serological markers of hepatitis B and correlates with high infectivity; in other words it identifies highly infectious individuals [4]. HBeAg is a non-structural protein produced by the actively replicating HBV and usually detec early in the serological course few months after infection.

Very limited data are available on the seroprevalence of HBeAg among Ghanaians. The presence of HBsAg neither represents viral replication nor infectivity. Therefore, the aim of the study was to determine the HBeAg status of asymptomatic HBsAg positive prospective blood donors at the KATH blood transfusion center in Kumasi, Ghana and to know whether HBeAg positivity modulated the levels of alanine aminotransferase. Data from the current study could influence policy on HBV vaccination and public education programmes of governments of resource limited countries to include compulsory HBV vaccine boosters for all citizens and increasing public education to levels comparable to that of HIV/AIDS. Also, this study could influence the policy on the laboratory diagnosis of HBV by informing clinicians and other stakeholders in resource limited health institutions to test the serological profile of HBV once a patient or an individual is tested positive for HBsAg.

## Methods

### Study design

A cross-sectional study was conducted to investigate the sero-prevalence of HBeAg infection among asymptomatic prospective blood donors with HBV infection at the KATH blood transfusion centre in Kumasi, Ghana. One hundred and fifty (150) participants were randomly recruited for the study at the KATH during the period of August 2012 to April 2013. Each participant completed a standard questionnaire, which included demographic information, history of blood transfusion, type of donor, history of unprotected sex, number of sexual partners etc.

### Ethical clearance

Ethical clearance for the study was obtained from the committee on Human Research Publication and Ethics from the Kwame Nkrumah University of Science and Technology (KNUST). Informed consent was obtained from each prospective blood donor recruited into the study. Participants who met the blood donation criteria and tested positive for HBsAg were included in the study.

### Experimental procedures

The serum samples were initially screened at the Transfusion Medicine Unit of the KATH for HBsAg, using Accu-Tell strips (AccuBioTech/China) according to the manufacturer′s instructions and transported to KNUST for further investigations. The HBV Rapid Test Kits (Egens, ISO9001, ISO13485, CE; HS Code: 38220010) is a lateral flow chromatographic immunoassay; was employed for the qualitative detection of HBsAg and HBeAg, in serum/plasma according to the manufacturer's instructions. The alanine aminotransferase activity in each of the samples was determined with Selectra ProS^®^ biochemistry auto analyser (Vital Scientific BV, Dieren-The Netherlands) with reagents from ELITech^®^ clinical systems according to the manufacturer's instructions.

### Statistical analyses

Data analysis was performed using SPSS version 20. Categorical and continuous variable were compared using chi square and student t test respectively The differences were considered to be statistically when p value obtained was <0.05.

## Results

One hundred and fifty prospective blood donors who tested positive to the HBsAg during screening at the KATH blood transfusion centre participated in this cross-sectional study. The study participants comprised of 116 (77.3%) males and 34 (22.7%) females with a ratio of approximately 3:1. Their ages ranged from 16 to 59 years with a mean of 25.35 ± 8.77. Twenty (20) of the study participants were found to be HBeAg-positive giving an overall prevalence of 13.3% of which 18 (15.5%) were males and 2 (5.9%) were females (p = 0.146).

The rate of seropositivity of HBeAg was categorized according to age groups (Table 1).

**Table 1:** Distribution of HBeAg seropositvity and seronegativity according to age groups

Age group (years)	HBeAg Positive		
	n/N (%)	95% CI	*P* value
10-20	12/60 (20.00)	11.68 - 31.93	
21-30	6/61 (9.84)	4.25 - 20.19	
31-40	2/16 (12.50)	2.24 - 37.28	
41-50	0/8 (0.00)	0.00 - 37.22	
≥ 51	0/5 (0.00)	0.00 - 48.95	
**Total**	20/150 (13.33)	8.73 - 19.77	0.291

Data are presented as numbers or percentage. CI is calculated at 95%. P-value is significant at p<0.05. HBeAg = Hepatitis B e antigen, CI = Confidence interval. Pearson Chi-Square= 4.963, df=4, P=0.291

ALT activity investigation among the study subjects showed that 117 of them had normal levels out of which 13 (11.1%) were HBeAg positive and 104 (88.9%) were HBeAg negative. Of the remaining 33 who had elevated levels of ALT, 7 (21.2%) were HBeAg positive and 26 (78.8%) were HBeAg negative. There was no statistical difference between the ALT activity in HBeAg positive individuals and that in HBeAg negative individuals, p = 0.132 (Table 2).

**Table 2:** Alanine aminotransferase in HBeAg positive and HBeAg negative group

ALT( IU/L)	HBeAg		Total
	Positive	Negative	
Normal (≤40)	13 (11.1%)	104 (88.9%)	117 (100%)
Elevated(>40)	7 (21.2%)	26 (78.8%)	33 (100%)
**Total**	20 (13.3%)	130 (86.7%)	150 (100%)

Pearson Chi-Square=2.273 df =1 P=0.132

## Discussion

The current study showed a seroprevalence rate of 13.3% of HBeAg among asymptomatic prospective blood donors who visited the KATH blood transfusion centre in Kumasi, Ghana. Our finding reflects a pool of individuals who may be highly infectious and serve in sustaining viral transmission and evolution in the population of the Ashanti region of Ghana, suggesting that the future burden of liver cancer or HCC associated with HBV is likely to be high.

Lower seroprevalence rates of HBeAg-positive hepatitis compared to the current study have been reported in various parts of the southern region of Nigeria. For example; Akinbami and colleagues studied a total number of 267 HBsAg-positive blood donors and reported a seroprevalence of 8.2% [5]. Also, Otegbayo and colleagues reported 10.8% prevalence among blood donors in Ibadan [6]. Studies by Akinbami's group, Otegbayo's group and the current study demonstrate that HBeAg-positive individuals within the West African sub region may range from approximately 8-13%. These useful findings would help policy makers and researchers in West Africa where hepatitis B infection is high to direct resources aimed at reducing HBV infection. A significant proportion of the current study population (86.7%) was HBeAg-negative, consistent with a reported similar high value of 86.4% by Sagnelli and colleagues from Italy [7]. These findings suggest HBeAg-negative hepatitis B infection is highly prevalent among individuals worldwide.

The current study revealed that with advancing age, spontaneous seroconversion takes place leading to a reduction in the frequency of HBeAg (Table 1) and an increase in anti-HBe antibody (data not shown). That is probably why an increasing age dependent reduction in the level of HBeAg seroprevalence was reported (Table 1). This report is supported by studies from Japan, which showed that the prevalence of HBeAg positivity decreased with increasing age [8].

The elevated levels of aminotransferase correlate highly with ongoing chronic hepatitis, which begins the sequalae of complications. However the current study shows no statistical difference between the HBeAg positive individuals and HBeAg negative individuals with respect to ALT levels among the study population ( 2). The result is in line with a study that showed that 40-50% of all HBeAg-positive patients may have normal ALT levels for prolonged periods [9]. Although serum ALT is used as a biochemical marker in assessing hepatic injury, its elevation does not always correlate with disease severity [9]. Recent studies have showed that an elevated ALT does not accurately predict significant liver injury and that commencing antiviral therapy should not be heavily based on a particular ALT threshold [10].

In the current study, the upper limit of serum ALT reference range was 40 IU/L as recommended by the manufacturer of the reagents used. It must however be noted that reference ranges may vary between geographic locations and ethnic groups.

The limitation to the current study is the small sample size. Another challenge was that a large proportion of the study population was HBeAg-negative which, might have been due to HBV variants that are unable to produce high amounts of the secreted protein that bears the ‘e’ antigen epitope due to mutations in the precore or core promoter regions of the HBV genome.

## Conclusion

The 13.3% prevalence of HBeAg among HBV chronic carriers of Ghanaian blood donors suggests an expansion and maintenance of a reservoir for chronic HBV infection with a public health “time bomb“ waiting to explode. Although vaccination against HBV in West Africa is compulsory for children there is the need for governments to initiate policies to follow up vaccinees to boost their antibodies to HBV once the vaccine antibodies have waned.
